# The finger loop of the SRA domain in the E3 ligase UHRF1 is a regulator of ubiquitin targeting and is required for the maintenance of DNA methylation

**DOI:** 10.1074/jbc.RA119.010160

**Published:** 2019-09-03

**Authors:** Robert M. Vaughan, Scott B. Rothbart, Bradley M. Dickson

**Affiliations:** Center for Epigenetics, Van Andel Research Institute, Grand Rapids, Michigan 49503

**Keywords:** DNA-binding protein, E3 ubiquitin ligase, epigenetics, molecular dynamics, DNA methylation, allosteric regulation, SRA domain, string method in collective variables, ubiquitin-like with PHD and RING finger domains 1 (UHRF1)

## Abstract

The Su(var)3–9, enhancer of zeste, and trithorax (SET) and really interesting new gene (RING) finger–associated (SRA) protein domain is conserved across bacteria and eukaryota and coordinates extrahelical or “flipped” DNA bases. A functional SRA domain is required for ubiquitin-like with PHD and RING finger domains 1 (UHRF1) E3 ubiquitin ligase activity toward histone H3, a mechanism for recruiting the DNA methylation maintenance enzyme DNA methyltransferase 1 (DNMT1). The SRA domain supports UHRF1 oncogenic activity in colon cancer cells, highlighting that UHRF1 SRA antagonism could be a cancer therapeutic strategy. Here we used molecular dynamics simulations, DNA binding assays, *in vitro* ubiquitination reactions, and DNA methylation analysis to identify the SRA finger loop as a regulator of UHRF1 ubiquitin targeting and DNA methylation maintenance. A chimeric UHRF1 (finger swap) with diminished E3 ligase activity toward nucleosomal histones, despite tighter binding to unmodified or asymmetric or symmetrically methylated DNA, uncouples DNA affinity from regulation of E3 ligase activity. Our model suggests that SRA domains sample DNA bases through flipping in the presence or absence of a cytosine modification and that specific interactions of the SRA finger loop with DNA are required for downstream host protein function. Our findings provide insight into allosteric regulation of UHRF1 E3 ligase activity, suggesting that UHRF1's SRA finger loop regulates its conformation and function.

## Introduction

Extrahelical or “flipped” cytosine bases are a distinct feature of structurally resolved complexes of DNA bound to endonucleases (1, 2), thymine DNA glycosylase (3), DNA methyltransferases (4, 5), protein methyltransferases (6–8), and the E3 ubiquitin ligases UHRF1 and UHRF2 (9–12). Of the resolved DNA-binding domains that coordinate flipped bases, Su(var)3–9, enhancer of zeste, and trithorax (SET) and really interesting new gene (RING) finger–associated (SRA)^3^ domains are promiscuous readers of cytosine nucleotides modified at the C5 position by methylation (5mC), hydroxymethylation (5hmC), formylation (5fC), and carboxylation (5caC) (7, 13 and Table 1). SRA domains (also named YDG domains) are annotated in over 4,000 proteins across bacteria and eukaryota (14). In mammals, SRA domains are found in only two proteins (15), the DNA methylation maintenance factor UHRF1 and the structurally related but functionally distinct UHRF2 (16, 17).

**Table 1:**
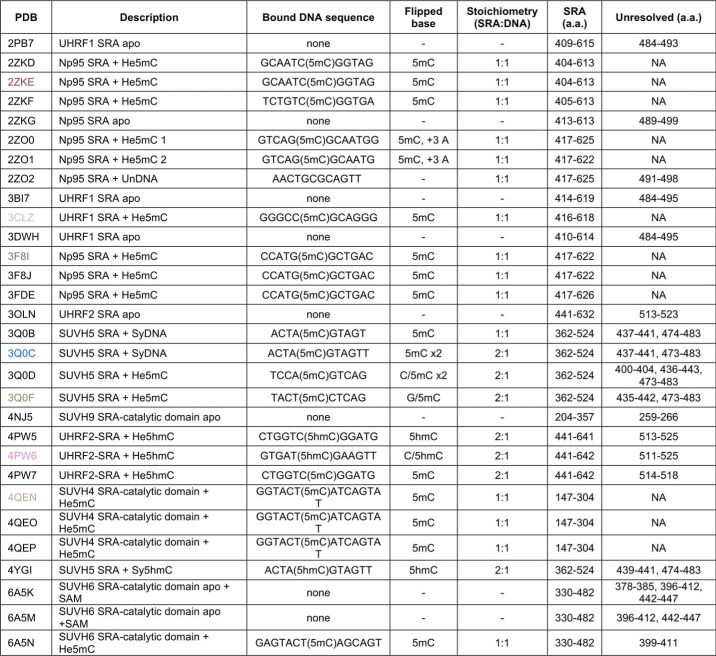
**Summary of structural data for resolved SRA domains** Colored PDB IDs correspond to the bound DNA as represented in Fig. 1*C*.

A functional UHRF1 SRA domain, as evaluated by point mutations that disrupt DNA interaction, is required for sustaining the oncogenic activity of UHRF1 in colon cancer cells (18), adding to the motivation of UHRF1 SRA antagonism as a cancer therapeutic strategy (19). Further, a functional SRA domain is required for UHRF1 E3 ubiquitin ligase activity toward histones (20, 21), a recruitment mechanism for DNMT1 (22).

**Figure 1.**
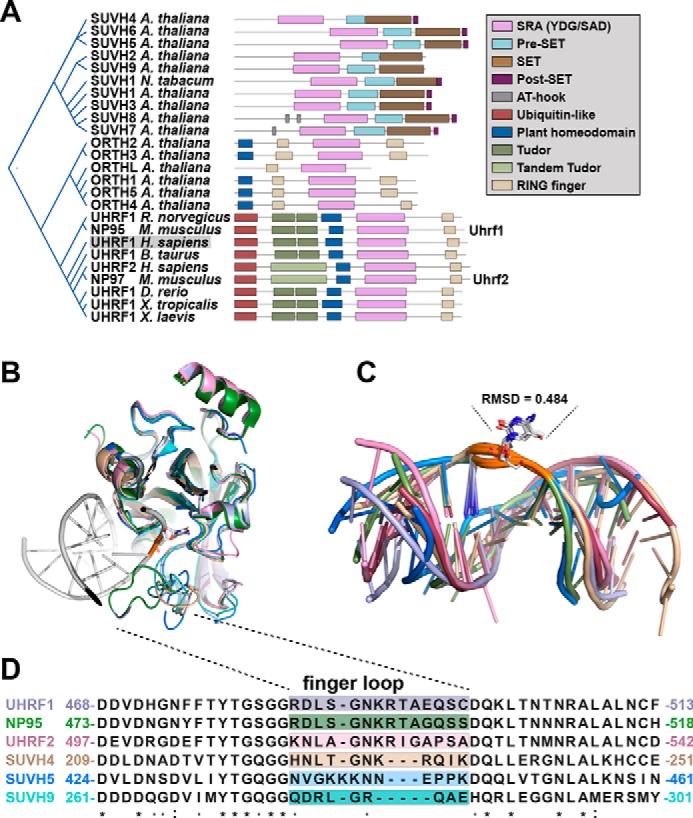
**Structural analysis of SRA domains reveals divergent finger loops.** *A*, phylogenetic analysis and domain maps of proteins that have annotated SRA domains in reviewed UniProt entries. *B*, all-atom structural alignment of SRA domains (colored as in *D*; PDB codes 2ZKD, 3CLZ, 3Q0C, 4NJ5, 4QEN, and 4PW6), performed by align command in PyMOL. For simplicity, only hemimethylated DNA (He5mC) bound to UHRF1 SRA is shown (PDB code 3CLZ). *C*, overlay of SRA-bound DNA molecules after alignment by all-protein atoms using the align command in PyMOL (colors correspond to Table 1); the root mean square deviation was calculated only for the shared atoms of the flipped 5-methylcytosine and 5-hydroxymethylcytosine. *D*, amino acids surrounding SRA finger loops aligned with ClustalW.

Here we sought to understand the mechanics of DNA binding and base flipping by the UHRF1 SRA domain through application of molecular dynamics simulations. The free energy measurements supported a model where base flipping was independent of the SRA finger loop, a defining element of SRA domains, consistent with the variety of extrahelical bases found in structural studies (Table 1). We next evaluated how the finger loop of the UHRF1 SRA domain influenced binding to modified DNA or E3 ligase activity. Notably, we found that a mutant UHRF1 (finger loop swapped from SUVH5 SRA, *Arabidopsis thaliana* protein methyltransferase) was an inactive E3 ligase toward nucleosomal histones despite tighter binding to unmodified, asymmetric, and symmetrically methylated DNA oligonucleotides. Finally, we demonstrate that the finger loop of UHRF1 is required for the maintenance of DNA methylation. Our studies lend insight into allosteric regulation of UHRF1 E3 ligase activity and support a model where the SRA finger loop serves as an important regulator of UHRF1 conformation and function. Because of the conservation of the core structure of the SRA domain (Fig. 1*B*), the findings from this study are likely applicable to other SRA domain–containing proteins.

## Results

### Structural analysis of SRA domains

We began our study by evaluating the homology across various SRA domains. Phylogenetic clustering segregated UniProt-reviewed SRA domains by their known or putative enzymatic functions as SET domain–containing methyltransferases or RING-containing E3 ubiquitin ligases (Fig. 1*A*). Comparison of available models of DNA–SRA costructures revealed a high degree of conservation in the core SRA domain structure (Fig. 1*B*). The position of the flipped DNA base was nearly identical for all SRA domains included in this analysis, with a total root mean square deviation of 0.484 for atoms shared between 5mC and 5hmC after alignment (Fig. 1*C*). Notably, the majority of contacts between the SRA and DNA were through the phosphate backbone of DNA. An NKR-containing “finger” loop, so named for an asparagine, lysine, and arginine (NKR) motif in UHRF1 that hydrogen-bonds with the Watson–Crick pair opposite the flipped base (9), was divergent across SRA domains (Fig. 1*D*). We observed that resolution of the finger loop was associated with SRA-to-DNA stoichiometry; finger loops were only resolved when 1:1 binding was observed (Table 1). The flexible finger loop (*i.e.* unresolved in crystallography) was a common feature of SRA domains bound to symmetrically modified DNA, as noted previously (12).

Collectively, these data associated changes in SRA–DNA interactions with unique sequence compositions of SRA finger loops. First, the finger loop is likely involved in selective binding to modified cytosines (12). Second, the lack of finger-loop resolution for some DNA-bound SRA domains in crystallography points to flexibility. These data led us to hypothesize that the finger loop is dispensable for base flipping but important for the SRA's selective binding to modified DNA.

### Base flipping is facilitated through distortion of the phosphate DNA backbone

**Figure 2.**
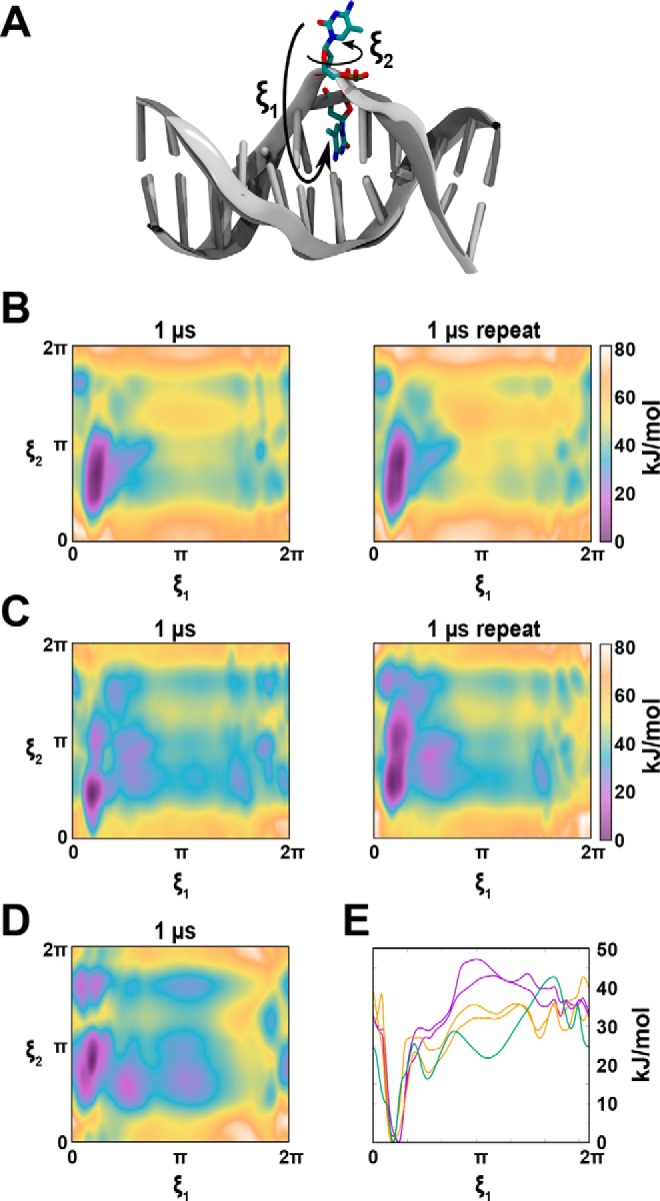
**Constraining the DNA phosphate backbone destabilizes base pairing and lowers the energy barrier to spontaneous base flipping.** *A*, overlaid structural models of intrahelical (*background*) and extrahelical (*foreground*) 5-methylcytosine; the extrahelical position is the start of the simulation and is the bound pose from the UHRF1 SRA–He5mC complex (PDB code 3CLZ). ξ_1_ indicates rotation of the nucleoside subunit around the phosphate backbone of 5-methylcytosine as plotted in *B–E*. ξ_2_ measures rotation of the base around the sugar linkage of 5-methylcytosine as plotted in *B–E. B–D*, free-energy plots of 1-μs molecular dynamics simulations of He5mC in *A* that is unrestrained (*B*), 5′ and 3′ phosphates surrounding 5-methylcytosine restrained to match PDB code 3CLZ (*C*), or restrained at all backbone phosphates (*D*). *E*, ξ_1_ rotation of 5-methyl-cytosine plotted against free energy from the simulations in *B* (*purple*), *C* (*yellow*), and *D* (*green*).

A prominent feature of DNA-bound SRA domains was a “puckering” of the DNA backbone surrounding the flipped base (Fig. 2*A*). This pucker results from a change in the angle of phosphodiester bonds that link deoxyribose sugar molecules in DNA. We speculated that distortion of the DNA backbone was a key aspect of base flipping.

To understand the mechanics of base flipping, we performed a series of 1-μs adaptively biased molecular dynamics simulations. The simulations were designed to systematically evaluate how DNA backbone restraint, as induced by interaction with SRA domains, contributed to the free energy of base flipping. Simulations were performed on 12-bp dsDNA with a single, hemimethylated CpG dinucleotide (He5mC) (Fig. 2*A*). The simulations are presented here as heatmaps of free energy with respect to the angles of rotation ζ_1_, the rotation of the nucleoside subunit around the phosphate backbone of 5mC, and ξ_2_, the rotation of the base around the sugar linkage (Fig. 2*A*). The simulations were performed under increasing levels of constraint on the DNA backbone, designed to mimic the interaction with an SRA domain. The three paradigms evaluated were with unrestrained He5mC (Fig. 2*B*), a restrained dihedral (23) in the backbone surrounding 5mC (Fig. 2*C*), or with all backbone phosphates restrained in the bound pose (Fig. 2*D*) from the UHRF1 SRA–DNA model (PDB code 3CLZ). Comparison of a single component (ζ_1_) across all simulations revealed that restraint of the entire DNA backbone (Fig. 2*E*, *green curve*), like that induced by SRA binding, created a lower-energy state observed near ζ_1_ = π or stabilized the flipped-base state of the DNA. Together, these simulations demonstrated that the extrahelical, base-flipped state of DNA can be stabilized by as much as 20 kJ/mol through manipulation of the DNA conformation and, notably, in the absence of the finger loop.

### DNA backbone rearrangement and SRA thumb insertion are energetic barriers to base flipping

**Figure 3.**
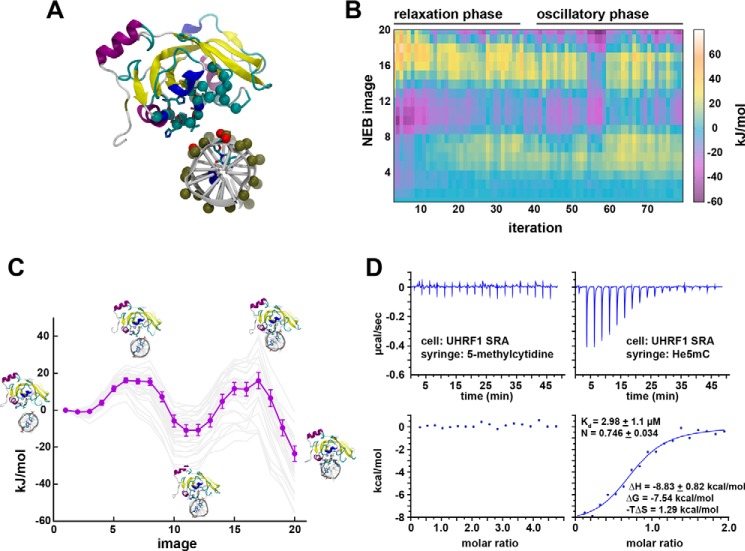
**Organization of the phosphate backbone and insertion of the SRA thumb are energetic barriers in the SRA–DNA interaction.** *A*, unbound starting pose for the string method in collective variables of the interaction between UHRF1 SRA Δ finger loop and He5mC (Δ finger loop; residues 484–494 were removed, leaving a Gly^483^-Gln^495^ peptide bond in its place). UHRF1 bound to He5mC (PDB code 3CLZ) served as the structural starting point for the simulation. *Spheres* indicate atoms included in the collective variables that were required to find their bound pose. *B*, free-energy plot of the string method (*image 1*, unbound; *image 20*, bound) over 80 iterations. *C*, representation of free energy and representative structural models over the oscillatory phase in the string method from iterations 50 to 79. 5-Methylcytosine and its paired guanosine are shown as *sticks* in the structural models. Each iteration is shown in *light gray*, whereas the average is shown in *purple*; *error bars* represent the 95% confidence interval. *D*, representative isothermal titration calorimetry result (*n* = 3), measuring the interaction between MBP-tagged UHRF1 SRA (35 μm) and 5-methylcytidine (1 mm, *left*) or He5mC (430 μm, *right*).

Our simulations performed on free DNA demonstrated that restraining the DNA backbone was sufficient to stabilize an extrahelical base. Although the SRA finger loop is implicated in the action of base flipping (8), these data suggest that base flipping is partially enabled by distortion of the phosphate backbone, a structural change that is unlikely influenced by the finger loop. We next employed the string method in collective variables (24) to determine whether the UHRF1 SRA domain can flip bases in the absence of its finger loop. The pathway from the apo SRA to DNA-bound SRA was optimized with respect to the collective variables, shown as *spheres* in Fig. 3*A*. The DNA-bound state was taken as the structurally determined pose of the UHRF1 SRA–He5mC complex (9) (image 20 in Fig 3*C*). The simulation iteratively estimated the free energy gradient at each of the 20 discrete snapshots along the path and moved each snapshot downhill in the direction orthogonal to the path (Fig. 3, *B* and *C*). After roughly 20 iterations, the optimized path can be seen to oscillate with no net decrease in free energy (Fig. 3*C*). The low-energy state characteristic of all paths in the oscillatory phase, beginning at image 10 in Fig. 3*C*, was driven primarily by the generation of contacts between the SRA domain and the negatively charged phosphate backbone of DNA. The large energy cost to arrive at image 17 in Fig. 3*C* was a result of insertion of the structurally conserved “thumb” (9) in the minor groove of DNA. Compared with Fig. 2*E*, this barrier for thumb insertion is roughly 5 kJ/mol less than the barrier to break base-pairing which suggests the thumb catalyzes eviction of the base from the paired geometry. These calculations (Fig. 3*B*) were performed in the absence of a finger loop and demonstrated that major energy barriers to SRA–DNA interaction were in the rearrangement of the DNA phosphate backbone and insertion of the thumb loop. Consistent with a binding model where the primary interactions between SRA and DNA are driven by phosphate backbone contacts, we detected no interaction between the UHRF1 SRA and 5-methylcytidine by isothermal titration calorimetry (ITC) (Fig. 3*D*, *left*). We also note that the stoichiometry between UHRF1 SRA and He5mC routinely fell between 0.5–1.0 in our ITC experiments (Fig. 3*D*, *right*), suggesting that UHRF1 may have 1:1 and/or 2:1 binding with DNA. This mixed stoichiometry is consistent with our model of the SRA domain as a promiscuous base flipper (or flipper of all bases), with specificity for the flipped base encoded by the finger loop (9).

Together, these data suggest that the SRA finger loop is not required for base flipping, consistent with previous suggestions that its primary role is in selective binding to modified cytosines (12). Additionally, the primary SRA–DNA interactions were through the phosphate backbone and the DNA-binding cleft of the SRA domain. Toward antagonizing the DNA binding activity of UHRF1 SRA, a 5mC analog may not be sufficient, as no binding was observed to free 5-methylcytidine.

### SRA finger loops interrogate modified DNA states

To evaluate the role of the finger loop in the regulation of both DNA binding and enzymatic activity, we generated a mutant UHRF1 where we swapped the finger from the well-characterized SUVH5 protein from *A. thaliana*. UHRF1 and SUVH5 have distinct finger loops (Fig. 1*D*) and distinct preferences for modified cytosines. UHRF1 has highest affinity for He5mC (25) and SUVH5 for symmetrically methylated (Sy5mC) and symmetrically hydroxymethylated DNA (Sy5hmC) (13). Additionally, UHRF1 enzymatic activity is allosterically regulated by DNA binding (20), whereas SUVH5 enzymatic activity is uncoupled from DNA binding (8). Because of the aforementioned observations, we reasoned that the UHRF1 SRA would acquire DNA binding characteristics of SUVH5 and that the finger swap would affect the enzymatic activity of UHRF1.

**Figure 4.**
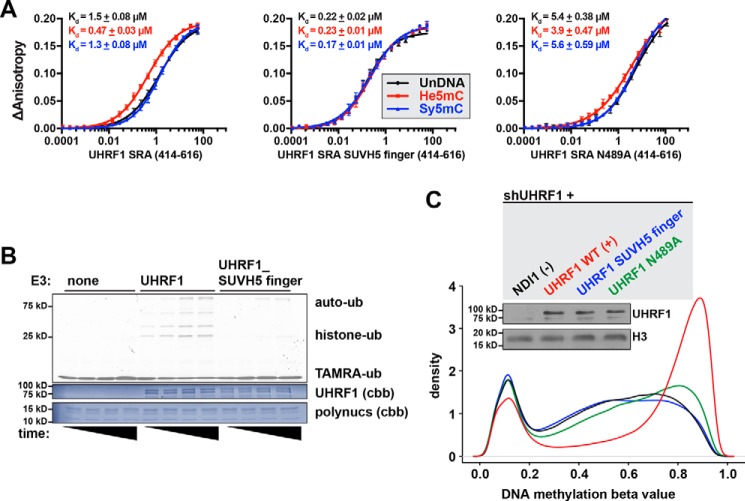
**The UHRF1 SRA finger loop controls selective binding to modified DNA and E3 ligase activity.** *A*, fluorescence polarization binding assays measuring the interaction between MBP-tagged UHRF1 SRA (*left panel*) or UHRF1 SRA that has residues 484–497 (see *colored box* in Fig. 1*D*) replaced with residues 434–445 from the SUVH5 SRA (*right panel*) and a FAM-labeled 12-bp dsDNA probe containing a single, centrally located CpG that was either unmodified (*UnDNA*), hemimethylated (*He5mC*), or symmetrically methylated (*Sy5mC*). Data were fit to a one-site binding model with the Hill slope, and *K_d_* is presented as ± S.E. of technical triplicate measurements (data shown are representative of two independent experiments). *Right panel*, SDS-PAGE followed by Coomassie Brilliant Blue staining of recombinant SRA domains. *B*, *in vitro* ubiquitination of purified HeLa polynucleosomes by either WT or SUVH5 finger-swapped, full-length UHRF1. Shown is a representative gel imaged for TAMRA-labeled ubiquitin after SDS-PAGE at 2, 5, 15, and 25 min (*n* = 2). *None*, control reactions containing all of the ubiquitin machinery and substrate without E3; *cbb*, Coomassie Brilliant Blue. *C*, density plot of Infinium MethylationEPIC BeadChip analysis of HCT116 cells 11 days after simultaneous knockdown of UHRF1 and transgenic cover by either NDI1 (− *control*), UHRF1 WT (+ *control*), UHRF1 SUVH5 finger, or UHRF1 N489A (β values: 0, unmethylated; 1, methylated). Also shown is a Western blot of HCT116 cells used in the DNA methylation analysis.

In fluorescence polarization binding assays, UHRF1 SRA with the SUVH5 finger bound tightly to DNA, irrespective of cytosine modification, with dissociation constants of ∼200 nm (Fig. 4*A*). Notably, this interaction was tighter than any of the WT UHRF1 SRA–DNA interactions measured. We conclude from these data that specificity for modified (or unmodified) DNA is encoded in the sequence of the SRA finger loop.

As the core structures of the UHRF1 and SUVH5 SRA domains are nearly identical (Fig. 1*B*), we posit that the finger loop must be a defining feature toward unique protein behavior. Consistent with this hypothesis, swapping the UHRF1 finger with the SUVH5 finger greatly diminished UHRF1 ubiquitin ligase activity toward HeLa polynucleosome substrates (Fig. 4*B*). This demonstrated that affinity for DNA and ubiquitin targeting were separable functions and were each dependent on the finger loop.

### The UHRF1 finger loop is required for DNA methylation maintenance

To evaluate the role of the finger loop in the regulation of DNA methylation maintenance, we used a genetic complementation system, essentially as described previously (18). HCT116 colorectal cancer cell lines were simultaneously transduced with shRNA against UHRF1 and rescued with either NDI1 (unrelated yeast control gene), UHRF1 WT (positive control), UHRF1 SUVH5 finger, or UHRF1 N489A. Antibiotics were used to select for dually transduced cells, and DNA was harvested 11 days after transduction. Western blotting confirmed UHRF1 knockdown and rescue (Fig. 4*C*). DNA methylation was evaluated at roughly 640,000 CpGs by Infinium MethylationEPIC BeadChip after quality control in SeSAMe (26). Only the UHRF1 WT rescue was able to maintain DNA methylation levels (Fig. 4*C*). The SUVH5 finger–swapped and N489A UHRF1 mutants were unable to maintain DNA methylation, phenocopying the control NDI1 rescue.

Here we used molecular dynamics simulations to demonstrate that base flipping by SRA domains is enabled by distortion of the DNA phosphate backbone and that this conformational change is induced independent of the finger loop. We further demonstrated that the SRA finger loop is responsible for selective binding to modified cytosines, regulation of UHRF1 E3 ubiquitin ligase activity, and maintenance of DNA methylation. Collectively, these data support a model where the SRA domain finger loop is dispensable for base flipping but required for selective binding to modified cytosines and subsequent regulation of host protein function.

## Discussion

To better understand the mechanics of DNA interaction by SRA domains, we performed adaptively biased molecular dynamics simulations to measure the free energy of DNA base flipping. Our computed barrier to base-flip free DNA matches previous reports (27). We observed 10- to 20-kJ/mol stabilization of the extrahelical base conformation (Fig. 2) when restraints were added to the DNA backbone to match the SRA-bound crystal structures. This observation is consistent with a mechanism where SRA domains sample a variety of bases through flipping, despite its modification, or lack thereof. When the proper base is flipped or not flipped, as encoded by the specific interactions of the finger loop with DNA, the downstream effect of the SRA-DNA interaction is recognized.

Our analysis of published SRA–DNA complexes revealed a variety of extrahelical bases, bound DNA sequences, and SRA:DNA stoichiometries (Table 1). In addition, we observed presumed “nonspecific” DNA poses, often involving the terminal bases of the oligonucleotide probes. To define “specific” DNA binding, we will consider the E3 ligases UHRF1 and UHRF2, whose enzymatic activity is linked to the specific recognition of He5mC or hemihydroxymethylated DNA (He5hmC), respectively (17, 20). The pose adopted by UHRF SRA domains and their asymmetrically modified cytosine is connected to their enzymatic function, but structural studies of UHRF1 and UHRF2 domains fail to inform on how specific cytosine recognition is linked to enzymatic activation. As the finger loop is often not resolved and is one of the most divergent regions across SRA domains, we hypothesize that the finger loop of SRA domains mediates host protein–specific function. We used the string method in collective variables to approximate the free energy difference of apo finger-less SRA to DNA-bound finger-less SRA to explore how DNA binding depends on the UHRF1 finger loop. Computations revealed that the binding event is spontaneous (negative in free energy) and that the cytosine is ejected from the apo base-paired conformation without the finger loop. The free energy difference was −23 kJ/mol (Fig. 3*C*). This value is 8 kJ/mol weaker than the empirically measured −31 kJ/mol for WT UHRF1 SRA (Fig. 3*D*). This is in agreement with the estimated finger loop contribution of 17 kJ/mol from Bianchi and Zangi (28). Consistent with our analysis of crystallographic data, our simulations suggest that the base-flipping mechanism does not require the UHRF1 finger loop. A reasonable hypothesis is that the finger loop regulates UHRF1 enzymatic activity through intramolecular contacts in a DNA-specific way. Together, either the finger loop of UHRF1 is involved in a conformation change that targets E3 to substrate, or the finger loop has an unanticipated role in the catalysis of E2 to target ubiquitin transfer.

UHRF1 antagonism is emerging as a therapeutic strategy in cancer (18, 19, 29–32). Recent genetic studies suggest that inhibition of the UHRF1 SRA–DNA interaction is a strategy for inhibiting tumor suppressor gene silencing in colon cancer cells (18). As we observed no detectable interaction between UHRF1 SRA and its natural ligand, 5-methylcytidine, our ITC data suggest that inhibitor scaffolds based on 5-methylcytidine alone may be insufficient. Rather, effective inhibitors of the SRA may need to engage a larger footprint beyond the hydrophobic pocket coordinating the flipped DNA base.

Efforts to evaluate the cellular and molecular functions of UHRF1 DNA binding have relied in part on point mutations that abolish DNA binding (18, 20). These mutations (G448D, N489A) lack precision to determine the contribution of He5mC-specific binding to UHRF1 function, as they indiscriminately decrease interaction with all DNA (Fig. 4*A*). The SUVH5 finger swap in the UHRF1 SRA represents a novel genetic manipulation that maintains high-affinity DNA interaction while perturbing the discrimination of cytosine modification states and disrupting E3 ligase function. This chimeric UHRF1 protein enabled precise dissection of the functional consequence of modified cytosine recognition by UHRF1 and supports a critical role of both specific DNA binding and E3 ligase activities of UHRF1 in the maintenance of DNA methylation.

## Experimental procedures

### Visualization of structural models and alignments

Existing structural models (PDB accession indicated throughout) were visualized in either PyMol or VMD. Structural alignments of SRA domains were performed using the align command in PyMol. Sequence alignments were performed using ClustalW through the EBI server. Phylogenetic analysis was performed using W-IQ-TREE (33) and visualized using iTOL (34).

### Free energy of DNA base flipping

Free energy of base flipping in free DNA was computed using fABMACS (35). The alanine dipeptide module of fABMACS was repurposed to include a pseudodihedral (23). Simulations were initially solvated with TIP3P (transferable intermolecular potential with 3 points) water and 150 mm NaCl, equilibrated by steepest descent for 5,000 steps, relaxed for 0.1 ns, and equilibrated to atmospheric conditions in the isothermal–isobaric ensemble (NPT) for 5 ns within GROMACS (36). Free energy of base flipping was computed in the canonical ensemble (NVT) using the modified adaptive biasing potential parameters: c = 0.01, b = 0.9, α = 8. Free energy simulations were computed over 1-μs trajectories.

### String method in collective variables

The minimum free energy path bridging from an unbound fingerless SRA (UHRF1 residues 416–613 were used with residues 484–494 removed, forming a new peptide backbone between Gly^483^ and Gln^495^) to a DNA-bound fingerless SRA was optimized with the string method in collective variables (24). The collective variables were taken as the Cartesian coordinates of the atoms, shown as balls in Fig. 3*A*. The bound-state configuration was taken from the X-ray structure (PDB code 3CLZ). The path was discretized into 20 images, and each image was harmonically restrained to its position with a spring constant of 8 kT/Å^2^. Each image used 1 ns of trajectory to compute the average free energy gradient before string updates. A total of 77 updates were made, and the path settled into a valley after about 50 updates. As Fig. 3*B* shows, the path oscillates for the last 20 iterations, and this is expected, given that the free energy gradient is estimated on 1 ns of sampling. The path optimization amounts to a total of 1.5 μs of simulation. The restraint was implemented within fABMACS (35), and the string updates were handled through bash scripts following the update rules from Ref. 24. Prior to path optimization, images were energy-minimized by solvation with TIP3P water and 150 mm NaCl, steepest descent for 5,000 steps, relaxed for 0.1 ns, and equilibrated to atmospheric conditions in the isothermal–isobaric ensemble (NPT) for 5 ns within GROMACS (36).

### Generation of recombinant proteins

UHRF1 SRA (residues 414–616, UniProt numbering) was cloned into a modified pQE vector as an N-terminal His_6_–maltose-binding protein (MBP) fusion. UHRF1 with the SUVH5 finger loop, as indicated in Fig. 1*D* (UHRF1 SRA residues 484–497 replaced with residues 434–445 from the SUVH5 SRA), was introduced by PCR-based mutagenesis. Proteins were expressed and purified as described previously (17). SRA domains in this study were characterized as MBP fusions, as we found that mutant SRA domains were less stable than the WT version after cleavage of the MBP tag. Full-length UHRF1 (1–793) or SUVH5 finger swap was purified as an MBP fusion and cleaved prior to use in *in vitro* ubiquitination reactions.

### Isothermal titration calorimetry

Binding measurements were performed on a MicroCal PEAQ ITC (Malvern) at 25 °C. UHRF1 MBP–SRA was dialyzed overnight at 4 °C in 25 mm HEPES, 100 mm NaCl, and 1 mm DTT. The next morning, 5-methylcytidine (Sigma, M4254) or annealed He5mC (sense, CCATG(5mC)GCTGAC; antisense, GTCAGCGCATGG), was resuspended in the same dialysis buffer as the SRA domain. UHRF1 SRA (35 μm) was loaded into the cell, and 5-methylcytidine (1 mm) or He5mC (430 μm) was loaded into the syringe. Nineteen injections (2 μl each, separated by 150 s) were performed after an initial injection of 0.4 μl.

### Fluorescence polarization DNA binding assays

Fluorescence polarization binding assays were performed essentially as described previously (17). Briefly, binding assays were performed in black 384-well plates in 25-μl volumes (25 mm HEPES (pH 7.5), 100 mm NaCl, 0.05% NP-40, 10 nm FAM–DNA oligonucleotide). DNA oligonucleotides were ordered from Eurofins Genomics and annealed to make either unmodified (sense, CCATGCGCTGAC; antisense, FAM-GTCAGCGCATGG), He5mC (sense, CCATG(5mC)GCTGAC; antisense, FAM-GTCAGCGCATGG), or Sy5mC (sense, CCATG(5mC)GCTGAC; antisense, FAM-GTCAG(5mC)GCATGG).

### In vitro ubiquitination reactions

Ubiquitination reactions were performed in 50 mm HEPES (pH 7.5), 66 mm NaCl, 2.5 mm MgCl_2_, 2.5 mm DTT with 50 nm E1 (UBE1, Boston Biochem, E-305), 666 nm E2 (UBE2D1, a gift from Dr. Joseph Harrison), 1.5 μm E3, 5 μm TAMRA–ubiquitin (BioVision, 7552), 0.5 μm HeLa polynucleosomes (EpiCypher, 16-0003), and 8 mm adenosine triphosphate (Sigma). Reactions were quenched by addition of SDS-PAGE loading buffer with fresh β-mercaptoethanol to reduce thioester ubiquitin conjugates, separated by SDS-PAGE, and imaged in-gel by Cy3 fluorescence (Azure c400).

### HCT116 UHRF1 rescue assay

To knock down endogenous UHRF1, a lentivirus with shRNA against UHRF1 (pLKO-PGK-PuroR, TRCN0000273256) was generated in HEK293T cells according to standard protocol (Addgene). UHRF1 and mutants were cloned into the pMXs-IRES-blasticidin retroviral vector (a gift from David Sabatini, Addgene 72876) by EcoRI and XhoI restriction sites without an affinity tag; yeast NDI1 served as a control for overexpression of UHRF1. A retrovirus was generated in Phoenix-AMPHO cells (a gift from Xiaotian Zhang). HCT116 cells, maintained in McCoy's 5A with 10% FBS (Sigma, F0926) and 1% penicillin–streptomycin (Thermo, 15140122), were simultaneously transduced with shUHRF1 and either UHRF1 WT, UHRF1 SUVH5 finger, UHRF1 N489A, or NDI1 and placed under selection of puromycin (2 μg/ml, for 2 days) and blasticidin (5 μg/ml, for 7 days) 48 h later. Cells were never allowed to reach confluence (only an issue for WT rescue as it had a clear growth advantage over others) and were collected 11 days after transduction by scraping into cold PBS. Collected cells were split for either protein or DNA extraction.

### DNA methylation analysis

Cells were lysed in one volume of 10 mm Tris (pH 8.0), 1 mm EDTA, and 0.5% SDS and digested by 1 mg/ml proteinase K (Life Technologies, 25530-015) for 1 h at 55 °C. Nucleic acids were extracted with equal volumes of phenol, followed by phenol:chloroform:isoamyl alcohol (25:24:1) and then chloroform:isoamyl alcohol (24:1), always saving the aqueous phase. Nucleic acids were precipitated by addition of sodium acetate (pH 4.8) and cold ethanol, pelleted by centrifugation, washed with 70% ethanol, dried, and resuspended in 10 mm Tris (pH 8). Nucleic acids were digested with 1 μl of RNaseA/T1 (Thermo, EN0551) for 1 h at 37 °C. DNA was precipitated as above, resuspended in 10 mm Tris (pH 8), measured by Qubit fluorimetric assay, and given to the Van Andel Research Institute Genomics Core for analysis by Infinium MethylationEPIC BeadChip. Methylation β values were extracted from .idat files (GSE135802) by the command openSesame in the SeSAMe R package (26). Any probe not represented in every sample was masked (640,190 probes included in the analysis), and data were visualized by densityPlot in the minfi R package (37).

### Western blot analysis

Cells were lysed in 10 mm PIPES (pH 7.0), 300 mm sucrose, 100 mm NaCl, 3 mm MgCl_2_, and 0.1% Triton X-100 supplemented with 1 protease inhibitor tablet/20 ml (Roche, 11697498001) on ice for 20 min. Lysates were quantified by Bradford assay (Bio-Rad, 11697498001). 5 μg of total protein was separated by SDS-PAGE, transferred to a PVDF membrane (Amersham Biosciences, 10600023) by semidry transfer apparatus (Hoefer), blocked in blocking buffer (5% BSA (Sigma) in PBS with 0.1% Tween 20 (PBST)), incubated with primary antibodies (UHRF1, Cell Signaling Technology, 12387S, 1:1000; H3, EpiCyhper, 13-0001, 1:50,000) overnight at 4 °C in blocking buffer, washed three times for 5 min in PBST, incubated with anti-rabbit HRP antibody (GE Healthcare, NA934, 1:10,000) for 1 h at room temperature, washed three times for 5 min in PBST, exposed to ECL reagent (Amersham Biosciences, RPN2232), and imaged on film (Kodak).

## Author contributions

R. M. V. and B. M. D. conceptualization; R. M. V. and B. M. D. formal analysis; R. M. V. validation; R. M. V. and B. M. D. investigation; R. M. V. and B. M. D. visualization; R. M. V. and B. M. D. methodology; R. M. V. and B. M. D. writing-original draft; R. M. V., S. B. R., and B. M. D. writing-review and editing; S. B. R. resources; S. B. R. and B. M. D. supervision; S. B. R. funding acquisition; B. M. D. software.

## Footnotes

3 The abbreviations used are:

SRA: Su(var)3–9, enhancer of zeste, and trithorax and really interesting new gene finger–associated
ITC: isothermal titration calorimetry
MBP: maltose-binding protein
5mC: methylation
5hmC: hydroxymethylation
He5mC: hemimethylated
Sy5mC: symmetrically methylated
FAM: carboxyfluorescein
TAMRA: tetramethylrhodamine.
